# Characterization of melanin from *Exophiala mesophila* with the prospect of potential biotechnological applications

**DOI:** 10.3389/ffunb.2024.1390724

**Published:** 2024-05-15

**Authors:** Cristy Medina-Armijo, Ibraheem Yousef, Antonio Berná, Anna Puerta, Abraham Esteve-Núñez, Marc Viñas, Francesc X. Prenafeta-Boldú

**Affiliations:** ^1^ Program of Sustainability in Biosystems, Institute of Agrifood Research and Technology (IRTA), Caldes de Montbui, Catalonia, Spain; ^2^ Faculty of Pharmacy and Food Sciences, University of Barcelona, Catalonia, Spain; ^3^ MIRAS Beamline, ALBA Synchrotron Light Source, Cerdanyola del Vallés, Catalonia, Spain; ^4^ IMDEA WATER, Alcalá de Henares, Madrid, Spain; ^5^ Department of Chemical Engineering, Universidad de Alcalá, Alcalá de Henares, Madrid, Spain

**Keywords:** bioelectroconductivity, dihydroxynaphthalene, extremophilic black fungi, FTIR chemical profiling, fungal melanin, metal biosorption, natural biomaterials

## Abstract

**Introducion:**

Fungal melanin is an underexplored natural biomaterial of great biotechnological interest in different areas. This study investigated the physical, chemical, electrochemical, and metal-binding properties of melanin extracted from the metallotolerant black fungus *Exophiala mesophila* strain IRTA-M2-F10.

**Materials and methods:**

Specific inhibitory studies with tricyclazole and biochemical profiling of whole cells by synchrotron radiation-based Fourier-transform infrared spectral microscopy (SR-FTIRM) were performed. An optimized extraction protocol was implemented, and purified fungal melanin was characterized using an array of spectrophotometric techniques (UV-Vis, FTIR, and EPR) and by cyclic voltammetry (CV) experiments. The metal-binding capacity of melanin extracts was also assessed by using Cr(VI) as a model heavy metal.

**Results:**

Inhibitory studies indicated that 1,8-dihydroxynaphthalene may be the main precursor molecule of *E. mesophila* melanin (DHN-melanin). The biochemical characterization of fungal melanin extracts were benchmarked against those from two melanins comprising the precursor molecule L-3,4-dihydroxiphenylalanine (DOPA-melanin): extracts from the ink of the cephalopod *Sepia officinalis* and DOPA-melanin synthesized in the laboratory. The CV results of melanin extracts incubated with and without cell suspensions of the electroconductive bacterium *Geobacter sulfurreducens* were indicative of novel semiquinone/hydroquinone redox transformations specific for each melanin type. These interactions may play an important role in cation exchange for the adsorption of metals and in microbial interspecies electron transfer processes.

**Discussion:**

The obtained results provided further evidence for the DHN-nature of *E. mesophila* melanin. The FTIR profiling of melanin extracts exposed to Cr(VI), compared to unexposed melanin, resulted in useful information on the distinct surface-binding properties of fungal melanin. The parameters of the Langmuir and Freundlicht isotherms for the adsorption of Cr(VI) were determined and compared to bibliographic data. Altogether, the inherent properties of fungal melanin suggest its promising potential as a biomaterial for environmental applications.

## 1 Introduction

Recent advances in biotechnology have paved the way in finding alternative materials with well-characterized structures that can be engineered, and are biocompatible, biodegradable, and harmless to public health and the environment. Certain natural biomaterials are of great interest to engineers and materials scientists because they offer a wide range of exceptional physical and chemical properties that can be exploited for a variety of applications, including bioremediation, bioelectronics, pharmaceuticals, cosmetics, packaging and food (Roy and Rhim, 2021). Among these novel extractable biomaterials, melanin is emerging as a promising substance with diverse potential applications (Tran-Ly et al., 2020a; Roy and Rhim, 2021).

Melanin comprises a group of ancestral and functionally analogous dark polymeric pigments that have evolved across a high diversity of organisms, including bacteria, fungi, plants and animals (Eisenman and Casadevall, 2012). According to a broadly accepted classification based on the chemical structure of precursor molecules and macroscopic characteristics (Pralea et al., 2019), there are three major types of melanin: i) Brown-black eumelanin is biosynthesized from L-tyrosine, which is converted into L-3,4-dihydroxiphenylalanine (DOPA-melanin); these precursors are oxidized further to 5,6-dihydroxyindole and 5,6-dihydroxyindole-2-carboxylic acid as the building blocks of eumelanin. ii) Brown-red phaeomelanin is also produced from the precursor molecule L-tyrosine, but its biochemical pathway involves the addition of cysteine to form benzothiazine intermediates. iii) Allomelanin is a very heterogeneous group of nitrogen-free polymers that include pyomelanin (a red pigment found in bacteria and some fungi), which is obtained through the catabolic pathway of L-tyrosine. The biodegradation of L-tyrosine generates p-hydroxyphenylpyruvate, which is oxidized to homogentisic acid as the main building block of pyomelanin. Moreover, within this last group of allomelanins, most ascomycetes synthetize melanin through the polyketide pathway, using 1,8-dihydroxynaphthalene (DHN-melanin) as precursor. Instead, basidiomycetes generally produce eumelanin (DOPA-melanin) in a pathway resembling that of animal melanin biosynthesis. All melanin types are generally characterized by cross-like amorphous polymeric aromatic structures that contain different functional groups (carboxyl, phenolic, hydroxyl, and amine), depending on the chemical nature of precursor molecules, which are interlinked by short-distance non-hydrolyzable and strong carbon–carbon bonds (Prota, 1992).

The secondary structure of melanin is contributed by its polyelectrolyte nature due to the stacking of aromatic oligomers by bond π–π interaction (Díaz et al., 2005). This extended π-conjugation of melanin allows for some degree of delocalized electron movement within the melanin network, contributing to limited intrinsic semiconductivity across the molecule. This delocalization of electron density plays a fundamental role in stabilizing metal–ion complexes that enhance the chelation capacity of melanin (Panzella et al., 2013). This phenomenon might also promote redox interactions with the surrounding environment, such as the direct participation of melanin in interspecies electron transfer pathways, whereby electrons are exchanged between different syntrophic microorganisms (Chen et al., 2014).

In fungi, melanization is particularly conspicuous in the so-called black fungi, which are predominantly clustered in the orders Dothideales and Chaetothyriales (Ascomycota). Melanin from black fungi has primarily been ascribed to the DHN-melanin type (Eisenman and Casadevall, 2012). Fungal melanization occurs at the cell wall, but, in black fungi, melanin accumulates within membrane-bounded organelles (rounded/elongated shape up to 500 nm in diameter) referred to as melanosomes, analogous to those found in animal cells (D’Alba and Shawkey, 2019). While melanin is not essential for fungal growth and development, it provides protection against a variety of stressors, including UV and ionizing radiation (Dadachova et al., 2008), the presence of heavy metals (Medina-Armijo et al., 2024), and toxic organic compounds (Prenafeta-Boldú et al., 2019a). Melanin also acts as an antioxidant, neutralizing reactive oxygen species (ROS) that are produced in some of the above-mentioned stress conditions (Jacobson and Tinnell, 1993).

The use of black fungi has already been proposed for a variety of biotechnological applications, such as the biosorption of heavy metals (Medina-Armijo et al., 2024) and volatile aromatic hydrocarbons (Prenafeta-Boldú et al., 2019b). Melanin seems to play a fundamental role in binding and metabolizing these toxic molecules, as well as the secondary reactive products that might result from their metabolism. Hence, fungal melanin is a particularly attractive biomaterial due to its sustainable and scalable production, as well its relatively easy extraction and purification, when compared to that of plants and animals. It is also a low-cost and high-performance alternative to synthetic analogues. However, and in contrast to the existing literature on bacterial (Pavan et al., 2019) and animal melanins (D’Alba and Shawkey, 2019), the melanin of black fungi has yet to be studied in detail.

In a previous paper (Medina-Armijo et al., 2024), we verified the biosorption capacity and kinetics of hexavalent Chromium Cr(VI) by whole cells and melanin extracts from the metallotolerant black fungus *Exophiala mesophila* strain IRTA-M2-F10. The aim in this study is to gain a deeper understanding of the properties of melanin from black fungi, specifically regarding its electrochemical properties and the binding of metals. To this end, we implemented an array of advanced analytical techniques for the characterization of the physicochemical and electrochemical characteristics of melanin extracts from *E. mesophila*. These results were then benchmarked against DOPA-melanins from natural (ink of the cephalopod *Sepia officinalis*) and synthetic origins. Understanding the specific properties of melanin from black fungi might be of interest for developing new environmental biotechnologies.

## 2 Materials and methods

### 2.1 Strains and growth condition

The black fungus *Exophiala mesophila* strain IRTA-M2-F10 (culture collection at the Institute of Agrifood Research and Technology, Spain) isolated previously from biodeteriorated glued ceramics was selected the source of fungal melanin because of its tolerance to heavy metals and easy cultivation under laboratory conditions (Medina-Armijo et al., 2024). Active cultures of this fungus were routinely maintained on potato dextrose agar (PDA; Condalab, Spain) incubated at 25°C in the darkness. Ten agar pre-grown fungal colonies of 5 to 7 mm in diameter (after about three weeks of incubation) were transferred into 250 mL of liquid medium that contained macronutrients (4.5 g KH_2_PO_4_, 0.5 g K_2_HPO_4_, 2.0 g NH_4_Cl, and 0.1 mg MgSO_4_ 7H_2_O per liter), 2 mL of a stock solution of micronutrients (120 mg FeCl_3_, 50 mg H_3_BO_3_, 10 mg CuSO_4_ 5H_2_O, 10 mg KI, 45 mg MnSO_4_ · H_2_O, 20 mg Na_2_MoO_4_ H_2_O, 75 mg ZnSO_4_ H_2_O, 50 mg CoCl_2_ 6H_2_O, 20 mg AlK(SO_4_)_2_ 12H_2_O, 13.25 g CaCl_2_ H_2_O, and 10 g NaCl, per liter), and yeast extract (4 g L^−1^) as the carbon and energy source. The incubation of this liquid culture was performed under dark conditions at 25°C on a horizontal shaker (80 rpm) and was maintained for three weeks. Fungal biomass was then harvested by centrifugation (18 g for 20 min) and used immediately for melanin extraction.

### 2.2 Inhibition of fungal melanin biosynthesis

Tricyclazole (5-methyl-1,2,4-triazolo[3,4-b] benzothiazole) (abcr GmbH, Karlsruhe, Germany) was employed as a selective inhibitor of the DHN-melanin biosynthesis pathway, resulting in visible changes in the fungal biomass dematiaceous pigmentation. This approach builds upon previous studies (Butler et al., 2009; Suwannarach et al., 2019) with some modifications. Notably, tricyclazole treatment allows for the specific accumulation of the biochemical precursor to fungal melanin, known as 1,8-dihydroxynaphthalene (DHN). This approach offers a valuable tool for comparing the precursors of fungal melanin with those of other melanin types, such as DOPA-melanin. Inhibition assays were performed with liquid cultures of *E. mesophila*, incubated in batches of 250 mL, as described in the previous section. After autoclaving the culture medium, a solution of tricyclazole in absolute ethanol was added to the liquid medium up to a final concentration of 50 μg mL^−1^, according to Kumar et al. (2015) with some modifications. Culture batches were then inoculated with colony plugs from cultures of *E. mesophila* growing on PDA, and incubated in the dark under shaking conditions at 25°C. The same procedure was repeated on batches without tricyclazole and three independent replicates were performed for each treatment/control condition. After three weeks of incubation, fungal cells were harvested by centrifugation (10000 rpm, equivalent to 9391 g, for 5 min at 4°C). The pellet was rinsed with Millipore water and resuspended with 1% formalin (Sigma Aldrich, Saint Louis, USA) as the fixing agent, and the cells were kept at 4°C for a maximum of 7 days.

Synchrotron-based Fourier transform infrared microscopy (SR-FTIRM) measurements on fixed cells of *E. mesophila* exposed to tricyclazole were performed at the infrared beamline MIRAS of the ALBA-CELLS synchrotron light source, using a Hyperion 3000 microscope coupled to a Vertex 70 spectrometer (Bruker, Germany). The SR-FTIRM was operated with a 36× Schwarzschild magnification objective (NA=0.65). The microscope was equipped with a mercury cadmium telluride (MCT) detector of 50 μm. All spectra were obtained using a single masking aperture size 15 × 15 μm, covering the full cell size. Single point maps of individual cells were collected in the 4000-900 cm^−1^ mid infrared range at 4 cm^−1^ spectral resolution with 256 co-adds scans per spectrum. A control measurement with the same parameters was performed with unexposed fungal cells. A background on the CaF_2_ slides without cells, with 10 measurements repeated, was also included. The absorbance spectrum for tricyclazole-exposed and unexposed fungal cells was obtained by subtracting the background spectrum. The subtraction was done directly (automatically) by means of the OPUS 7.5 software (Bruker, Germany), according to (Martínez-Rovira et al., 2019). This program was also used to perform multivariate principal component analysis (PCA) and Savitzky–Golay second derivative of the averaged absorbance spectra on FTIR profiles.

### 2.3 Extraction and purification of melanin

Melanin extraction from pre-grown fungal biomass of *Exophiala mesophila* IRTA-M2-F10 was performed by adapting previously described methods based on acid hydrolysis (Selvakumar et al., 2008; Suwannarach et al., 2019). The fungal biomass was first resuspended with 1M NaOH (1:1.5 w/v biomass/NaOH) and then autoclaved for 20 min at 120 ± 1°C. The resulting suspension was centrifuged at 3220 g (4000 rpm) for 20 min, and the supernatant was collected and acidified with 6N HCL final concentration (1:1 v/v NaOH–melanin/HCl) to pH 2.5 for 12 h at 100°C, in order to induce the precipitation of melanin. Centrifugation at 3220 g for 20 min was then performed to collect the melanin precipitate, which was then washed three times with deionized water and centrifuged again at 3220 g for 15 min. The final melanin pellet was lyophilized at a pressure of 0.7 mBar at -50°C for 24 hours, resulting in a black powder that was kept at -20°C for further studies. In order to compare and characterize the fungal melanin extract, melanin was also extracted from the ink of sepia (*Sepia officinalis*), following the same protocol with a few differences in the washing steps. Sepia melanin was firstly washed once with chloroform and centrifuged at 3220 g for 15 min, to remove oily and protein substances, and then twice with deionized water, and centrifuged at 3220 g for 15 min each time. In addition, synthetic melanin from the polymerization of L-3,4-dihydroxyphenylalanine (DOPA-melanin, Sigma-Aldrich, United Kingdom) was also used for comparative purposes.

### 2.4 Physicochemical characterization of melanin

The morphology and particle size of melanin precipitates obtained from *E. mesophila* were observed using a scanning electron microscope (SEM) model JEOL JSM-5610 (brand, Japan). A sample of the melanin powdery extract was coated with a gold layer using a vacuum sputterer to increase the conductivity of the samples. The physical and chemical properties of melanin extracts were determined by following similar protocols to those described in previous studies (Selvakumar et al., 2008; Suwannarach et al., 2019). Melanin solubilization tests on organic solvents were performed with 99.9% methanol, absolute ethanol, 96% acetone, 99.9% acetonitrile, 99.8% benzene, 99.9% 1-butanol, and 99.5% 2-propanol. Precipitation tests were conducted under acid conditions using 3M of HCl and 1% FeCl_3_. Decolorization assays were performed with 30% H_2_O_2_ and 10% NaOCl. These experiments were also performed with sepia melanin extracts and synthetic DOPA-melanin, using 0.1 mg of melanin in 5 mL of solution. All used reagents were of analytical grade.

Fungal and non-fungal melanin samples were subjected to an array of spectroscopic analyses, similar to the methods described in previous studies (Selvakumar et al., 2008; Gonçalves et al., 2012). Melanin (0.5 mg) was dissolved in 10 mL of 0.01 M NaOH in a quartz cube and loaded onto a UV-Vis spectrophotometer (model EMC-11S UV brand, Germany). The UV–visible absorption spectrum of the melanin samples was scanned in the wavelength range of 200–750 nm, and the 0.01M NaOH solution was used as a reference blank. Electron paramagnetic resonance (EPR) spectra of powdery melanin samples were obtained with an X-Band (9.7 GHz) Bruker ELEXSYS E500 spectrometer equipped with an ST8911 microwave cavity and a variable temperature unit, a field frequency lock system ER 033 M and an NMR Gaussmeter ER 035 M (Bruker, Germany). The instrumental parameters of EPR analysis were set at 100 kHz modulation frequency, 1.0 G modulation amplitude, 0.498 mW microwave power, 9.872 GHz and 20.48 ms scan time.

FTIR spectroscopy is a well-established technique for characterizing complex melanin polymeric matrices by detecting specific functional groups and chemical bonds present in their structure (Pacelli et al., 2020; Elsayis et al., 2022). The analysis of fungal melanin was performed using a Hyperion 3000 microscope coupled to a Vertex 70 spectrometer (Bruker, Germany). Samples of melanin extracts (1 mg) were mixed with KBr dried powder (1:100) in an agate mortar and ground for a few minutes to break up melanin and KBr lumps. The transmittance spectrum was obtained with melanin samples exposed to Cr(VI) and using non-exposed samples as control. The spectra of Cr(VI)-exposed melanin were acquired by subtracting the reference spectra from non-exposed raw melanin (4000-600 cm^−1^) according to the method of Karimi et al. (2021). The subtraction was done directly (automatically) using the OPUS 7.5 software (Bruker, Germany).

### 2.5 Electrochemical characterization of melanin

The electrochemical properties of fungal melanin were compared to those of cephalopod melanin extracts and synthetic DOPA-melanin, in solutions prepared by adding 15 mL of NaOH (1M) to 0.1 g melanin powder upon sonication for 2 hours at 37°C. The resulting solution was diluted with deionized water (resistivity 18.0 MΩ·cm) until reaching a final concentration of 5 g L^−1^ with a pH of 7.0 ± 0.05. Then, 80 µL suspensions of the studied melanin types were used to coat screen-printed electrodes (SPE) using gold working electrodes and a Ag/AgCl reference electrode (DRP-220AT, DropSens, Asturias, Spain). Electrochemical assays were then performed immediately using a PC-controlled potentiostat (NEV4, Nanoelectra S.L., Alcalá de Henares, Spain) by adapting the methodology from González et al. (2011) and Estevez-Canales et al. (2015). To gain a deeper understanding of melanin’s redox properties and potential interactions with electroactive bacteria, cultures of *Geobacter sulfurreducens* (GS), strain ATCC 51573, were mixed with melanin and deposited onto gold SPEs. Cyclic voltammetry (CV) experiments were conducted at two different scan rates, 0.01 and 0.05 V s^−1^, and two potential windows were explored: from -1.00 V up to 0.50 V in the absence of GS, and from −0.80 up to 0.50 V in presence of GS. Anaerobic conditions were maintained, as elsewhere described (Estevez-Canales et al., 2015). GS was grown at 30°C in a freshwater medium containing the following mineral salts (per liter): 2.5 g NaHCO_3_, 0.25 g NH_4_Cl, 0.06 g NaH_2_PO_4_·H_2_O, and 0.1 g KCl. Additionally, the medium contained 0.024 g C_6_H_5_FeO_7_ (ferric citrate), 10 mL of a vitamins mix, and 10 mL of a trace mineral mix (Lovley and Phillips, 1988). Anaerobic conditions were kept by purging the culture medium with a gas mixture N_2_:CO_2_ (80:20) to remove oxygen and maintain the pH of the bicarbonate buffer at 7.0.

### 2.6 Heavy metal sorption experiment

Melanin extracts were resuspended in 50 mL Erlenmeyer flasks containing Cr(VI), added as potassium dichromate (K_2_Cr_2_O_7_; Scharlab ExpertQ^®^, Sentmenat, Spain), in concentrations ranging from 20 to 200 mg Cr^6+^ L^−1^, and incubated at 25°C on a shaker at 150 rpm for various times (4 to 72 hours). These experiments were performed using 0.3 g of melanin powder at pH 6.5. Each condition was assayed in triplicate. At the end of incubation, the suspensions were centrifuged (3220 g for 10 min) and filtered with a 0.45 µm filter before the Cr(VI) concentration was determined. The collected samples were acidified with sulfuric acid for the solubilization of Cr(VI), which then reacted with 0.05% of 1,5-diphenylcabazide (Sigma-Aldrich, St. Louis, MI, USA) (0.5 ml) to form a purple-violet complex. The concentration of Cr(VI) was measured using a spectrophotometer (model EMC-11S UV brand, Germany). Absorbance was measured at a wavelength (λ) of 540 nm. The sorption capacity (*q_e_*, in mg Cr(VI) per g of melanin in dry weight) was calculated through the following equation:


(1) $$q_{e}=\;\left[C_{o}-\;C_{e}\;\right]\cdot \frac{V}{m}\;$$

where *C_0_* is the initial concentration of chromium, *C_e_* is the concentration at equilibrium (mg L^−1^), V is the volume of the incubations (L), and *m* is the dry weight of the fungal melanin powder added as a sorbent (g-DW). The experimental data on the Cr(VI) adsorption (*q_e_* on Equation 1) was fitted to the Langmuir and Freundlich isotherm models. The Langmuir isotherm assumes that adsorption occurs at a monolayer of specific and homogeneous sites, and, once an adsorbate unit occupies a site, no further adsorption can occur on that particular site, according to the following formula:


(2) $$\;q_{e}=q_{max}\;\frac{K_{L}\;.\;C_{e}\;}{1+K_{L}.\;C_{e}}$$

The *K_L_* value is a constant related to the energy of sorption, *q_e_* (mg g^−1^) is the equilibrium biosorption capacity, *q_max_* (mg g^−1^) is the maximum adsorption capacity, and *C_e_* (mg L^−1^) is the final concentration in a certain interval time. The Freundlich adsorption isotherm is an empirical model of the equilibrium between the adsorbate uptakes per mass unit of the adsorbent:


(3) $${\text{q}}_{\text{e}}={\text{K}}_{\text{F}}.{\text{C}}_{\text{e}}^{1/n}$$

where *n* is a constant related to the efficiency of sorption and sorption energy, *K_F_* is a constant measuring adsorption capacity, *q_e_* is the amount of adsorbate removed per mass unit of melanin, and *C_e_* is the equilibrium concentration of the adsorbate in solution. Isotherm model fitting to experimental data was performed with the linearized versions of the Langmuir and Freundlich equations.

### 2.7 Software and statistical methods

Experiments on Cr(VI) removal capacity by fungal melanin were repeated thrice, and the results are presented as the average with standard deviation (± SD). The software OriginPro v9.3 (OriginLab Corporation, Northampton, MA, USA) was employed for the fitting of Equations (2) and (3) to experimental data, by minimizing the sum of squared errors (SSE). The same software was used for the visualization of cyclic voltammetry (CV) curves, and for the extraction of relevant peak values.

## 3 Results and discussion

### 3.1 Melanization inhibitory studies

**Figure 1:**
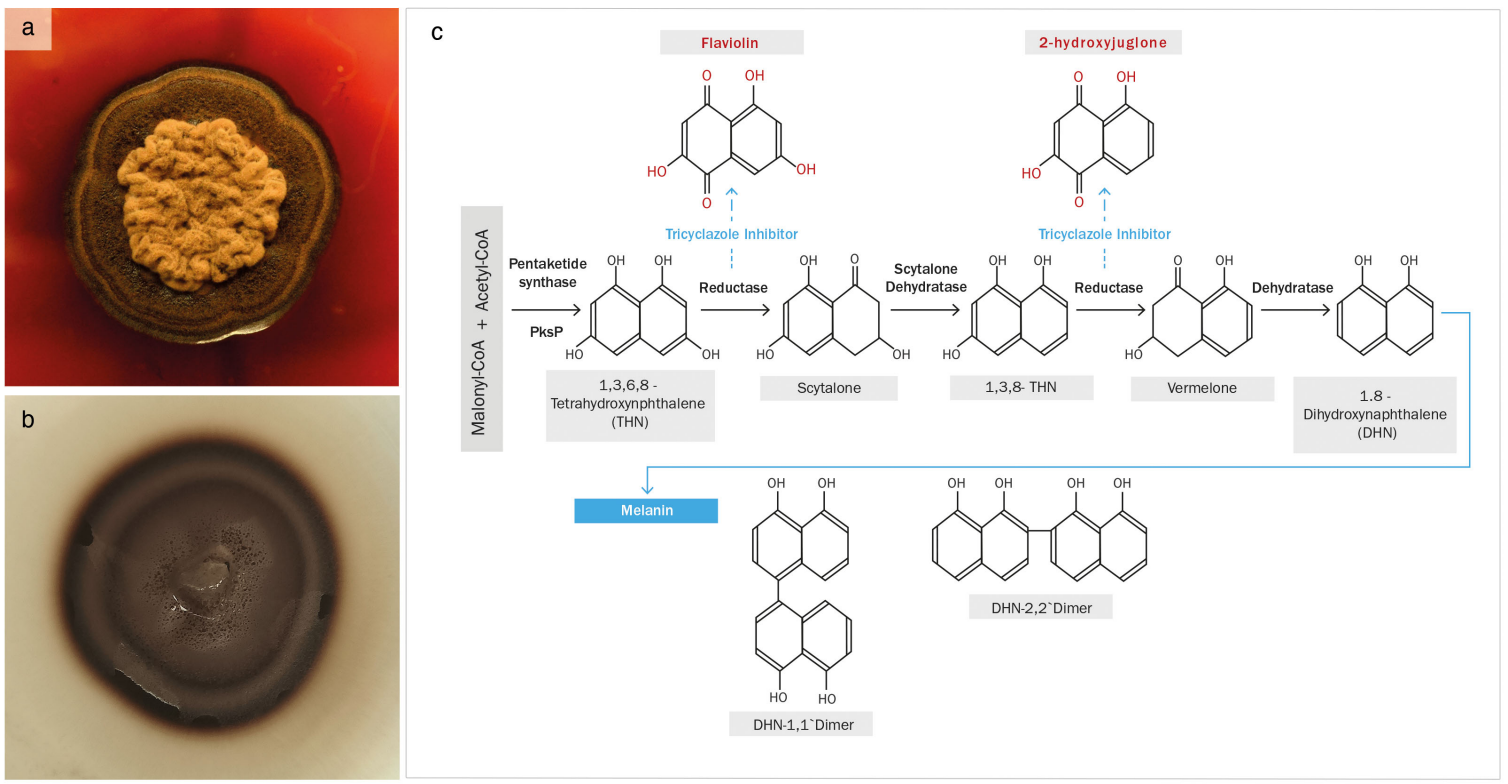
**(A)** *Exophiala mesophila* growing on PDA supplemented with 50 µg L^−1^ of tricyclazole, and **(B)** an analogous culture without tricyclazole; **(C)** proposed pathway for the biosynthesis of DHN-melanin, highlighting the tricyclazole inhibition of reductases that leads to the accumulation of the intermediates flaviolin and 2-hydroxyjuglone (Adapted from Lee et al., 2003).

Tricyclazole is a fungicide that is widely used in agriculture and other applications, as it has multiple modes of action, mainly by disrupting mitochondrial function and acting as a specific inhibitor of DHN-melanin biosynthetic pathway (Kong et al., 2018). An effective disruption of melanization was observed after exposing cultures of *E. mesophila* to tricyclazole for five days, both on plates (**Figure 1**) and in liquid media (not shown). The color of fungal cells shifted from black to reddish brown, and the agar medium was also tinged with a reddish color. This phenomenon has previously been attributed to the accumulation and excretion of the intermediates flaviolin and 2-hydroxy juglone, which are formed by the auto-oxidation of aromatic substrates when DHN-melanin biosynthesis is inhibited (Bárcena et al., 2015).

**Figure 2:**
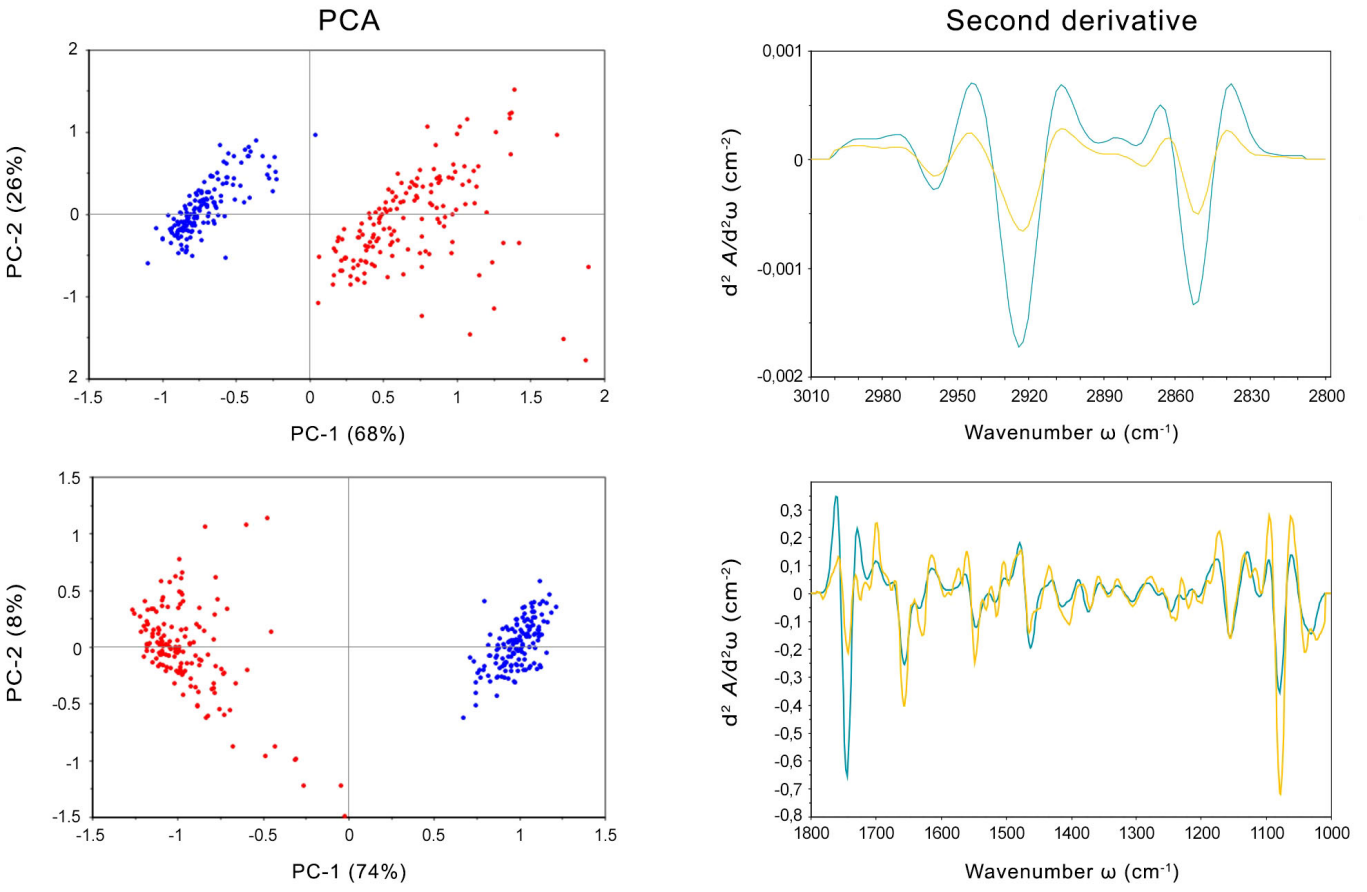
Left: PCA scores and loading plots of cells of *Exophiala mesophila* exposed to tricyclazole (T, red dots) and uninhibited cells (C, blue dots). Right: Savitzky–Golay second derivative of the averaged absorbance spectra in fungal cells exposed to tricyclazole (yellow lines) and uninhibited cells (blue lines). Up: results corresponding to the lipids’ (3000–2800 cm^−1^) spectral region. Down: results corresponding to the fingerprint (1800–1000 cm^−1^) spectral region.

SR-FTIRM was also employed to examine the intracellular response of *E. mesophila* to tricyclazole. Principal component analysis (PCA) was applied to identify clustering patterns in the biochemical profiles of fungal cells that were inhibited with tricyclazole (T) in comparison to non-exposed cells as control (C). **Figure 2** shows the PCA score loading plots and average FTIR spectra in the fingerprint and protein regions (1800-1000 cm^−1^), and in the high region (3000-2800 cm^−1^) dominated mainly by lipids. The changes induced by tricyclazole are clearly visible in both regions, and the PCA score plot shows that T and C FTIR profiles are well resolved. Clustering occurs mostly along the first axis, which explains 74% of the variance in the fingerprint region and 68% of the variance in the lipid region.

The second derivative of the FTIR spectra for T and C, along with the distribution of relative intensities for several spectral bands, was also performed (**Figure 2**). Notably, exposure to tricyclazole (T) triggered alterations in protein conformational structures associated with the amide I band at 1660 cm^−1^ (which corresponds to C=O stretching vibrations), the amide I band at 1638 cm^−1^ (N–H bending vibrations), and the amide II band at 1547 cm^−1^ (N–H bending and C–N stretching vibrations). These findings imply a shift in protein conformational structures from α-helix to β-sheet, as evidenced by the vibrational patterns (C=O, C–N, and N–H) associated with protein secondary structures (Martínez-Rovira et al., 2019). In addition, the PO^2−^ asymmetric band at 1242 cm^−1^ and the PO^2−^ symmetric band at 1087 cm^−1^, which are primarily derived from nucleic acids and the contribution of phospholipids, exhibit several alterations in intensity and peak position. This suggests that a range of variations in DNA organization occurred stemming from tricyclazole exposure on the cell. The spectra at 1120 and 1050 cm^−1^ were attributed to the symmetric C–C stretching of α-glucan and asymmetric C–O–C and P–O–C β-glucan complexes, respectively. These modifications in polysaccharide vibrations can be ascribed to changes in the cell wall. The distinct response of glucan could potentially impact the cell wall’s permeability, enhancing fungal metallotolerance. The lipid spectral region (3100–2800 cm^−1^) reveals alterations in the intensity of CH_2_ and CH_3_ symmetric and asymmetric stretching modes, which could be associated with the state of hydrocarbons and lipids unique to the cell wall. Hence, besides the inhibition of melanization, tricyclazole also affects the cell wall structure, potentially causing the trans-chains to become more rigid, perhaps as a protective mechanism against environmental stresses. This could, for instance, influence the transport of ions, water, and nutrients into the cell.

The impact of tricyclazole on melanin structure can also be inferred from the SR-FTIRM spectra (**Figure 2**). Peaks between 1670 and 1600 cm^−1^ correspond to conjugated double bonds C=C and C=O in the aromatic ring and C=O in secondary amine features of typical quinoid structures, like those found in melanins. The second derivative illustrates the reduction of these bands in cells exposed to tricyclazole. In other words, tricyclazole induced evident changes in the decreased intensity of peaks between 1670 and 1600 cm^−1^. This effect is attributed to tricyclazole’s inhibition of the polymerization of DHN, affecting the amount of melanin that accumulates in the cell.

Our research demonstrates the detrimental effects of tricyclazole on fungal growth, beyond the inhibition of melanization. This compound additionally alters the lipid composition of fungal cells, potentially leading to an increase in reactive oxygen species (ROS) levels through the peroxidation of lipid molecules, particularly those with methyl groups (Kumar et al., 2015). While tricyclazole at low doses may not be detrimental to cell survival, it can elevate ROS levels within cells, potentially affecting the integrity of various biochemical metabolic pathways. Hence, melanin inhibition may lead to DNA, lipid, and amide damage, among other potentially deleterious effects. Previous studies have emphasized melanin’s role in alleviating oxidative stress and neutralizing free radicals (Smith and Casadevall, 2019), and shown that the tricyclazole-mediated inhibition of melanin synthesis alters fungal growth and physiological processes (Bárcena et al., 2015). Tricyclazole inhibits enzymes that reduce 1,3,6,8-tetrahydroxynaphthalene to scytalone and 1,3,8-trihydroxynaphthalene to vermalone (**Figure 1**; Lee et al., 2003). This enzyme repression induces DNA transcription and methylation, which may be the main cause of the changes in the protein and DNA of melanin intermediates observed in this study, themselves related to the process of autoxidation (Zhan et al., 2016).

The FTIR profiling confirms the disruptive effect of tricyclazole on the biosynthesis of DHN-melanin in black fungi and, more specifically, the predominance of this melanin type in *Exophiala mesophila*. However, further research is warranted to elucidate the precise interplay between melanin production and tricyclazole’s mechanism of action. Additionally, investigations into the potential alterations of key physicochemical properties of the fungus in response to tricyclazole treatment are necessary.

### 3.2 Physicochemical characterization of fungal melanin

**Figure 3:**
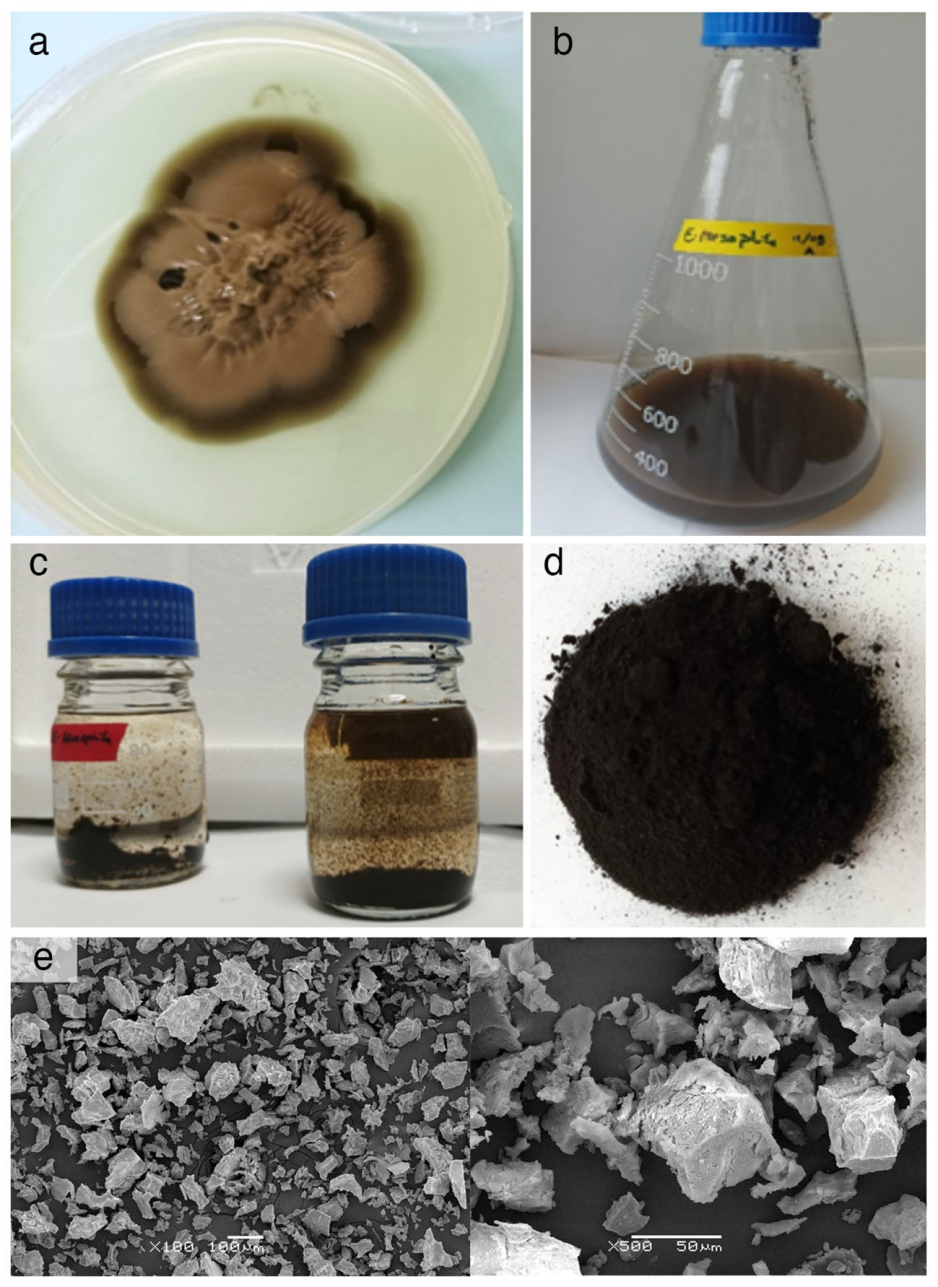
**(A)** PDA agar culture used as inoculum and **(B)** nutrient-rich liquid culture used to obtain larger amounts of biomass of *Exophiala mesophila*; **(C)** acid hydrolysis of fungal melanin after 12 hours at 100°C and precipitation of melanin in HCl at 6N; **(D)** lyophilized fungal melanin powder; **(E)** SEM microscopic images at different magnifications of melanin extracts.

The protocols implemented for the extraction and purification of melanin from the fungus *Exophiala mesophila* yielded 155.05 ± 25.64 mg (*n* = 4) of melanin extract per gram fungal biomass, both expressed on a dry weight basis. This extract had the macroscopic appearance of a powdery dark brown pigment (**Figure 3**). A more detailed morphological characterization through scanning electron microscopy showed that fungal melanin extracts consisted of an array of opaque and amorphous black granules of 10 to 200 µm in size (**Figure 3E**).

The results of the solubilization, precipitation, and oxidation tests are similar for the fungal and cephalopodal melanin extracts, as well as for the synthetic DOPA-melanin, and are also consistent with previous research (Gonçalves et al., 2012; Suwannarach et al., 2019). All three melanins were insoluble in distilled water, but were soluble in 1 M NaOH and 1 M KOH. They were also largely insoluble in most of the tested organic solvents (methanol, absolute ethanol, acetone, acetonitrile, benzene and chloroform), with the exception of methanol and absolute ethanol, in which they were slightly soluble. The precipitation test was positive with 2N HCl and 1% FeCl_3_ solutions, where a brown precipitate was clearly observed. All three melanins were also effectively decolorized with 30% H_2_O_2_ and 10% NaOCl solutions.

**Figure 4:**
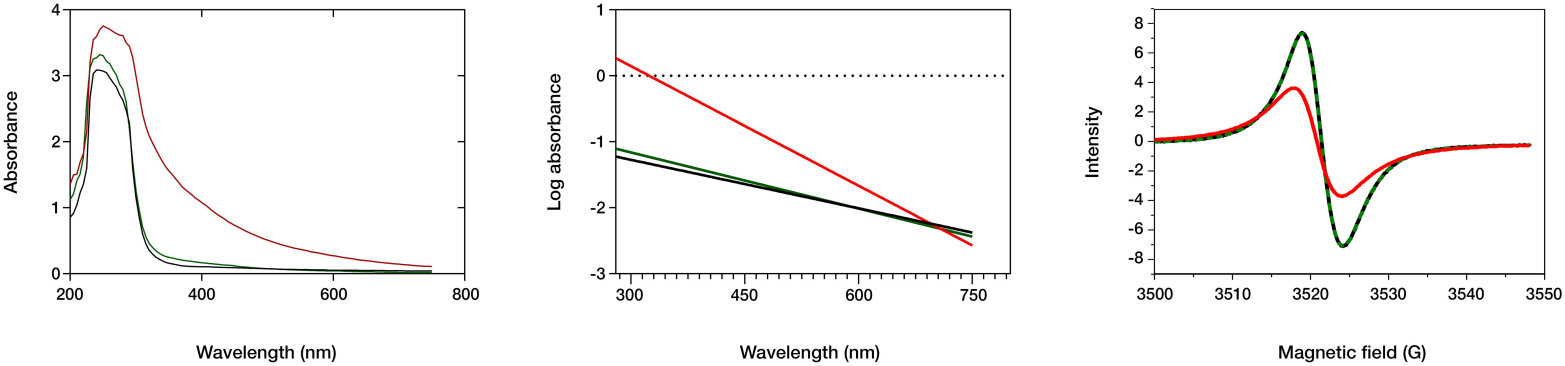
Spectral analysis of fungal (black line) and cephalopodal (green) melanin extracts, and of synthetic DOPA-melanin (red). Left: UV and visible absorbance raw spectra. Center: log plot of the negative linear slope absorbance region against the wavelength. Right: EPR spectral analysis.

Melanin’s insolubility in most common solvents hinders its detailed chemical characterization. However, several analytical techniques offer rapid and informative results for melanin analysis, including ultraviolet–visible (UV-Vis) spectroscopy, electron paramagnetic resonance (EPR), and Fourier-transform infrared (FTIR) spectroscopy (Gonçalves et al., 2012; Suwannarach et al., 2019; Pacelli et al., 2020; Mostert, 2021). The spectral properties of three different melanin types were investigated using these techniques (**Figure 4**). The UV-Vis absorption spectra reveal similar maximum absorbance peaks for the natural fungal and cephalopodal melanin extracts, as well as the synthetic DOPA-melanin (wavelengths of 225, 236 and 224 nm, respectively), and sharp decreases in absorbance in the visible region. Similar profiles have been reported previously with melanin from various fungi, in which the maximum absorbance was observed in the UV range of 200−300 nm, swiftly decreasing toward the visible region (Suryanarayanan et al., 2004; Selvakumar et al., 2008; Eisenman and Casadevall, 2012; Gonçalves et al., 2012). The overall absorption profile of melanin has previously been modeled as multiple overlapping distinct chromophores (Di Mauro et al., 2017; Mostert, 2021), and high absorbance in the UV region generally reflects the abundance of aromatic rings containing conjugated double bonds (Kumar et al., 2011).

The log absorbance of melanin (**Figure 4**) against wavelength in the visible region (Δlog A/λ) has a typically linear correlation with a negative slope, which might be specific for every melanin type (Selvakumar et al., 2008; Pal et al., 2014; Suwannarach et al., 2019). Fungal and cephalopodal melanin extracts had very similar Δlog A/λ slopes of −0.0025 and −0.0028, respectively, but the synthetic DOPA-melanin showed a steeper slope of −0.0060. Previously reported Δlog A/λ slope values for the melanin of black fungi range from −0.0015 for *Phyllosticta capitalensis* (Suryanarayanan et al., 2004) to −0.0030 for *Exophiala pisciphila* (Zhan et al., 2011). The UV-Vis absorbance profile of melanin has been attributed to potential electronic transitions within individual chromophore components present in its aromatic polymeric structure (Meredith et al., 2006). Tran et al. (2006) suggested that the inherent chemical heterogeneity of melanin contributes to this phenomenon. The negative Δlog A/λ slope emphasizes the role of melanin’s chromophoric absorption at shorter wavelengths, with light scattering playing a minimal role, especially in this region of the spectrum (Di Mauro et al., 2017). These findings highlight the excellent photoprotective properties of melanin against UV and visible radiation and have paved the way for the development of novel applications in various fields, including UV-resistant textiles, UV exposure sensors, and biomedicine (Tran-Ly et al., 2020a).

Electron paramagnetic resonance (EPR) spectroscopy, a well-established technique for identifying free radicals in melanin (Enochs et al., 1993), offers insights into various aspects of its redox and magnetic properties. The g-value, a parameter derived from EPR spectra that reflects the ratio of magnetic moment to angular momentum in a magnetic field, provides valuable information for the identification of melanin. Analyses of fungal and cephalopodal melanin extracts and synthetic DOPA-melanin revealed similar g-values (2.0030, 2.0028, and 2.0033, respectively). Additionally, the EPR spectra exhibited peaks at a magnetic field strength of 3524 G (**Figure 4**), confirming the presence of free radicals, a hallmark characteristic of melanin (Zhan et al., 2011; Gonçalves et al., 2012; Suwannarach et al., 2019). EPR spectroscopy reveals a characteristic single line in the spectrum with slight asymmetry (**Figure 4**), which corresponds to a semiquinone radical formed by the coupling of a free electron with the aromatic rings within melanin. The EPR signal can be influenced by redox processes, particularly by changes in pH (Szpoganicz et al., 2002). Some studies suggest that redox reactions involving heavy metals modulate the EPR signal, possibly due to physical and magnetic interactions between the metals and melanin (Żądło and Sarna, 2019). However, discrepancies exist regarding the mechanisms of free radical production at varying redox potentials (Szpoganicz et al., 2002). Notably, at high pH, some studies report a potential depletion of quinones, which are precursors for semiquinone formation (Meredith et al., 2006).

**Figure 5:**
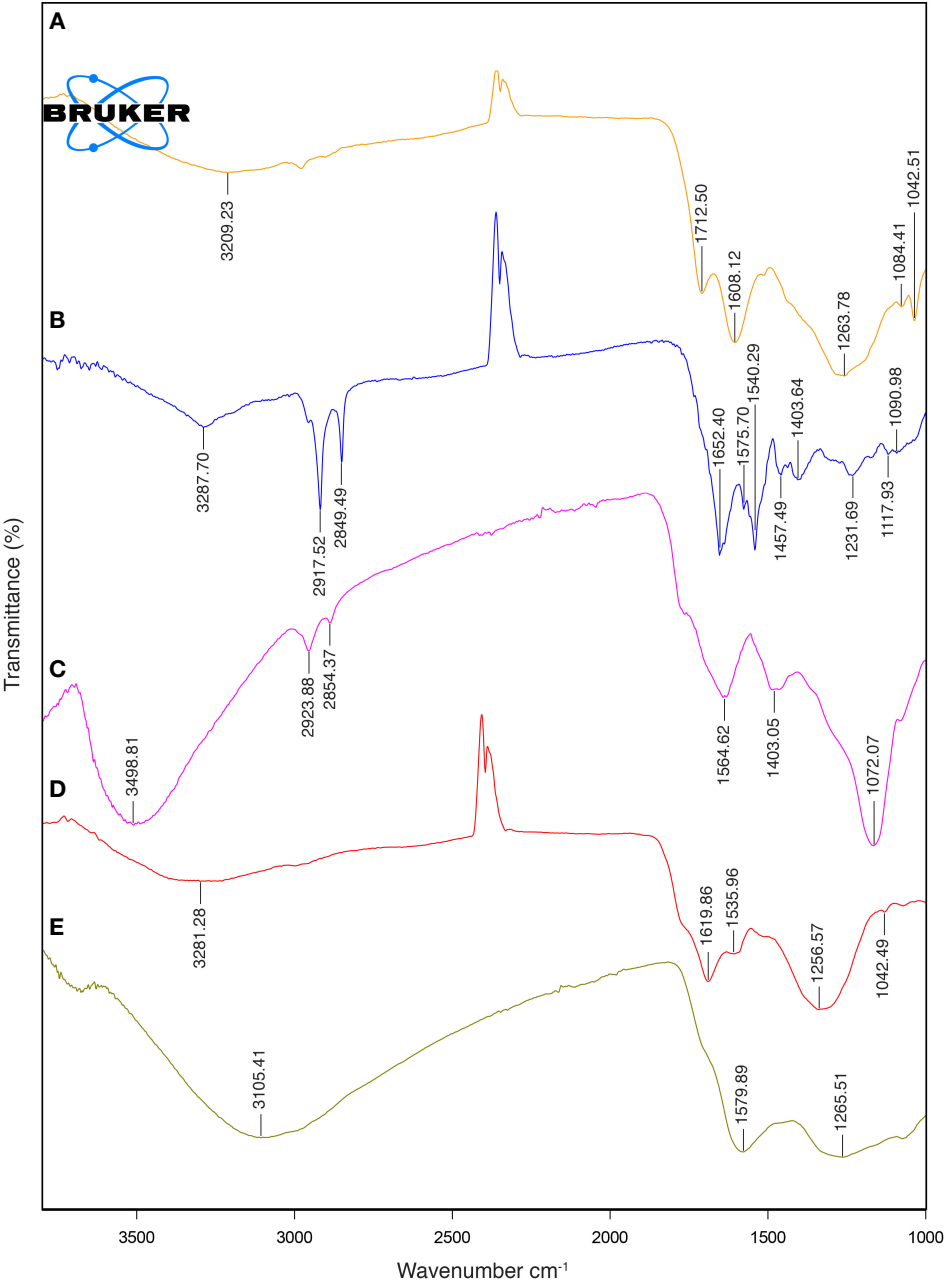
FTIR spectra: **(A)** synthetic DOPA-melanin, **(B)** natural fungal melanin, **(C)** natural fungal melanin/Cr(VI), **(D)** natural cephalopodal melanin and **(E)** natural cephalopodal melanin/Cr(VI).

The FTIR spectra of natural melanin extracts (both fungal and cephalopodal) and of synthetic DOPA-melanin are coherent with the different melanin types they are assigned to (**Figure 5**). This analysis showed a broad adsorption peak at 3300–3000 cm^−1^, which has been attributed to the stretching vibrations of the OH, NH, COO^-^ and phenolic groups that are characteristic of melanins in general (Barretto and Vootla, 2020). These broad bands peaked at 3288, 3281 and 3209 cm^−1^ for melanin extracts of *E. mesophila*, *S. officinalis*, and synthetic DOPA melanin, respectively. However, due to the peak overlap in this region, it is not possible to determine the exact contribution of each functional group. Interestingly, two high-intensity absorption bands at 2918–2849 cm^−1^ were observed exclusively with fungal melanin, which could be attributed to the vibration of aliphatic C–H stretching. Similar studies agree with these specificities of the FTIR profiling of fungal DHN-melanin (Pacelli et al., 2020; Elsayis et al., 2022) when compared to DOPA-melanin. In fact, the relatively lower abundance of aliphatic chains in DOPA-melanin, compared to DHN-melanin, could be a key distinction between these two melanin types.

The aromatic skeletal C=C ring stretching of amide I C=O and COO- groups was assigned at 1652–1620 cm^−1^ in fungal and cephalopodal melanins, while in synthetic DOPA-melanin, these functional groups were detected at 1608–1713 cm^−1^, as seen previously (Watt et al., 2009; Zhan et al., 2011). The aromatic/pyrrole stretching vibration and indole ring vibration were observed at 1540 cm^−1^ and 1536 cm^−1^ in the fungal and cephalopodal melanin, respectively. However, synthetic DOPA-melanin did not show vibrations in these functional groups, which finding is consistent with previous reports, in that synthetic melanins do not exhibit indole ring bands (Watt et al., 2009; Zhan et al., 2011). Phenol groups’ C–O or OH deformation of alcohol groups were characterized by peaks at 1232, 1257 and 1264 cm^−1^ in all melanin types. The presence of peaks near 1250 cm^-1^ has been reported in previous studies on melanin (Mbonyiryivuze et al., 2015; Suwannarach et al., 2019). The FTIR spectrum of *E. mesophila* melanin is similar to that of melanin extracted from other black fungi such as *Hortaea werneckii* (Elsayis et al., 2022) and *Spissiomyces endophytica* (Suwannarach et al., 2019). Overall, the observed FTIR features demonstrate a strong resemblance of the analyzed samples to the typical spectral profiles of the melanin types they are associated to, concerning OH or NH, and C=O stretching or the phenolic group (Pralea et al., 2019). The high abundance of functional groups in the melanin structure contributes to the interesting properties and potential applications of this biomaterial.

In **Table 1**, the main physicochemical characteristics of the melanin types analyzed in this study have been summarized and compared with similar data from previous studies. While natural melanins extracted from *E. mesophila* and *S. officinalis* display a very similar spectrophotometric behavior when analyzed with UV-Vis and EPR techniques (**Figure 4**), they are clearly distinct in their origin and chemical structure, as shown by the FTIR profiling (**Figure 5**). Synthetic DOPA-melanin, on the other hand, is also quite different from sepia melanin, despite sharing the same precursor molecule. Synthetic melanins are usually purer and, depending on the manufacturing method, have a more uniform but generally less complex structure than their natural counterpart. These results suggest that similar environmental pressures might have caused the development of independent origins of melanin throughout evolution, but caused a convergence on similar functions, such as protection against UV or the adsorption of toxic metals. Whether or not melanins have evolved independently multiple times, or share a common ancestry with subsequent diversification and convergence driven by environmental pressures, is a matter that deserves further research.

**Table 1:** Summary of the main physicochemical properties of different types of melanins.

Melanin type	Source/ Species	λ max (nm)	Δlog A/λ	EPR g-value	FTIR main peaks (cm^−1^)	Solubility	Reference
Fungal (DHN)	Biomass*/* *Exophiala mesophila*	225	-0,0025	2.003	3288 2918 1652 1540 1232	Insoluble: water, organic solvents Partly soluble: strong alkali, borate buffer	This study
	Biomass/ *Cryomyces antarcticus*	~215	-0.0027	2.004	2917 2843 1621 1024	Insoluble: water, organic solvents Soluble: strong alkali	Pacelli et al. (2020)
	Biomass/ *Hortaea werneckii*	240	n/a	n/a	3438 2927 1637 1240	Insoluble: water, most organic solvents Soluble: strong alkali, ethanol, methanol, dimethyl sulfoxide (upon vigorous mixing)	Elsayis et al. (2022)
Cephadophodal (DOPA)	Ink/ *Sepia officialis*	236	-0,0028	2.003	3281 1620 1536 1257	Insoluble: water, organic solvents Soluble: strong alkali, borate buffer	This study
		280	n/a	n/a	3100–3300 2920 1650 1200	n/a	Wang and Rhim (2019)
Bacterial (Untypified)	Biomass/ *Rhizobium radiobacter*	222	−0,0030	2.005	3277 2933 1514 1227	Insoluble: water, organic solvents, strong acids Soluble: strong alkali	Żądło and Sarna (2019)
Synthetic (DOPA)	Commercial	~215	n/a	2.004	3360–3000 1621	Insoluble: water, organic solvents Soluble: strong alkali	Pacelli et al. (2020)

λ max, frequency of the maximum UV-VIS absorbance; Δlog A/λ, log absorbance–wavelength slope; n/a, not available.

### 3.3 Electrochemical characterization of melanin

The electrochemical characterization of fungal and cephalopodal melanin extracts using cyclic voltammetry (CV) revealed their redox properties within a potential interval from -1.00 to +0.50 V *vs.* Ag/AgCl and at a scanning rate of 10 mV s^−1^ on a gold SPE electrode. The comparison of CV responses on gold electrodes and in the absence/presence of both natural melanins revealed the existence of electrochemical interactions. The presence of melanin completely modified the interface of the electrode/solution, blocking the characteristic electrochemical fingerprint response originating from the adsorption/desorption of hydroxide anions (Hamelin et al., 1990). The voltametric response of melanin-covered gold electrodes resembled the CV obtained by González et al. (2011). Anodic and cathodic peaks appeared at different potentials, mainly due to the different electrode materials (highly oriented pyrolytic graphite (HOPG) instead Au) and reference electrode (SPE instead of Ag) used.

**Figure 6:**
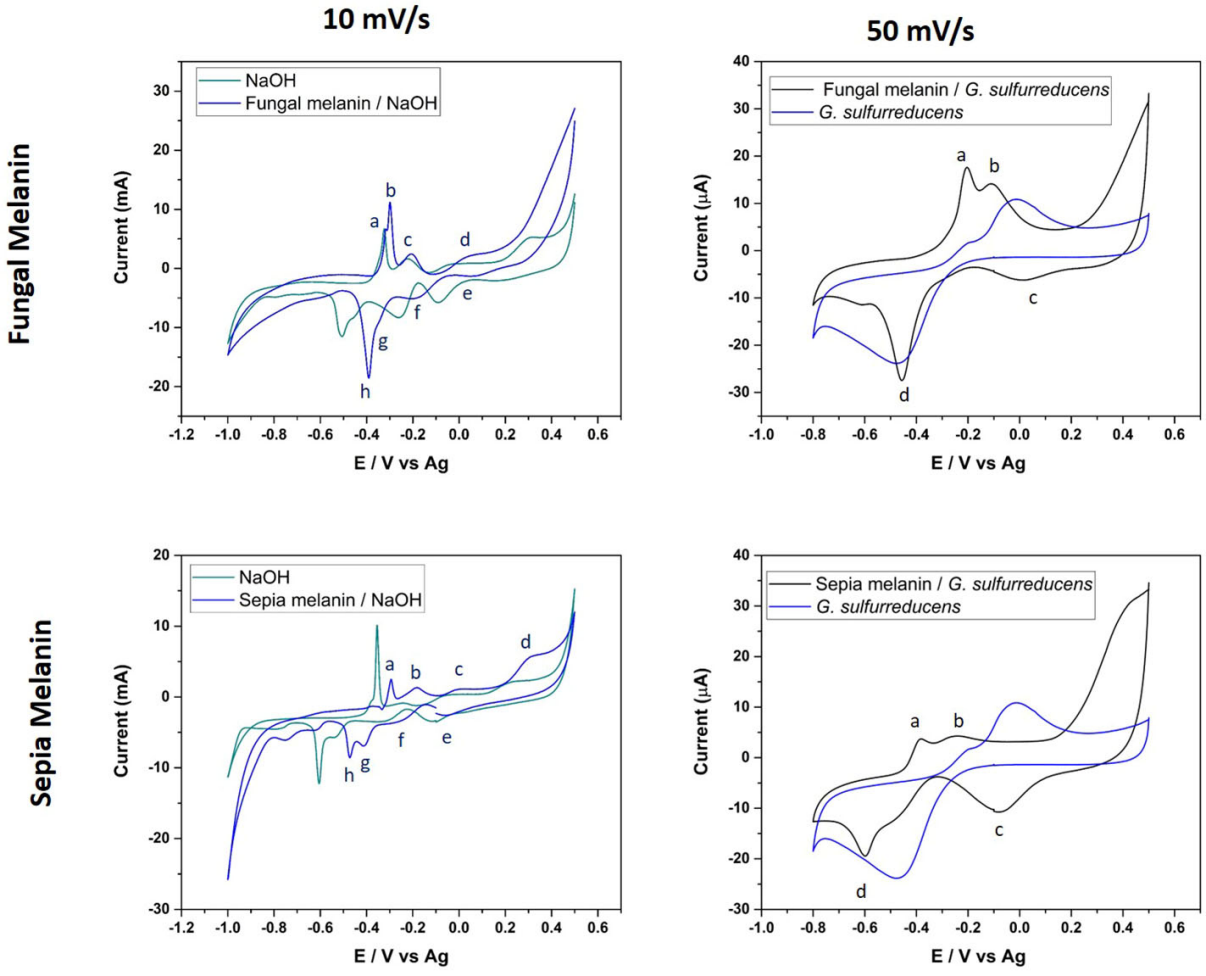
Cyclic voltammograms of fungal and cephalopodal melanin extracts, at a scan rate of 10 mV s^−1^ with 0.1 M NaOH, and a voltage range from −1.0 to +0.5. The scan rate 50 mV s^−1^ corresponds to natural fungal and cephalopodal melanin in contact with *G. sulfurreducens*, with a voltage range from −0.8 to 0.5 V.

Regarding the electrochemical properties of the studied melanins, the synthetic DOPA-melanin exhibited a rather simple CV profile, with two redox couples observed at −0.28 V (cathodic) and -0.40 V (anodic), and a reversible couple at -0.18 V (results not shown). Instead, the fungal and cephalopodal melanins (**Figure 6**) revealed a complex CV with four redox couples appearing at 0.06 V (peaks d/e), −0.20 V (peaks c/f), −0.30 V (anodic) and −0.35 V (cathodic) (peaks b/g), and −0.32 V (anodic) and −0.39 V (cathodic) (peaks a/h). Notably, two redox couples (d/e and c/f) exhibited a reversible behavior at a relatively strong positive potential. In contrast, the other two couples (a/h and b/g) displayed a non-reversible behavior at slight positive potentials (reductive peaks exhibited lower potentials in comparison to the oxidative peaks). The reversible redox couples can be assigned to oxidation/reduction processes involving quinone groups, which are typically present in melanin (Kumar et al., 2016), while the other two redox couples may correspond to processes related to the uptake of cations by melanin (Orive et al., 2009; González et al., 2011). It must be noted that the specific nature of both processes is highlighted by the different shapes of the voltametric peaks for each redox couple. The peaks associated with the oxidation/reduction of quinone groups are very broad, probably due to the different chemical environments surrounding these groups. In the case of the redox couples related to cation uptake, voltametric peaks are very sharp and narrow. There are slight differences between the CVs of fungal and cephalopodal melanin extracts, regarding the peak currents (**Figure 6**). The redox couple b/g of fungal melanin shows higher peak currents compared to the redox couple a/h. In the case of cephalopodal melanin, the peak current of the redox couple a/h is comparable to that of the redox couple b/g, probably revealing a different mechanism for cation uptake. This singular behavior opens the door to exploring the sensitivity of each melanin type to different cations in the uptake process.

To provide more compelling evidence for redox cycling and potential interactions of natural melanins, melanin extracts of both *E. mesophila* and *S. officinalis* ink were combined with viable cells of the electroactive bacterium *Geobacter sulfurreducens* (GS) on a gold SPE electrode. The recorded voltametric currents were larger for the combination of natural melanin plus GS than with GS alone (at 50 mV s^-1^), which points to the enhancement of extracellular electron transfer processes involving different compounds, such as quinones, catalyzed by the functional groups of melanins (Kumar et al., 2016). According to the CV of **Figure 6**, a broad anodic peak (b) appeared at −0.10 V in both melanin extracts, overlapping the potential at which the oxidation of quinone groups occurs. In the cathodic scans, broad peaks (d) were observed at −0.45 V and −0.6 V for fungal and cephalopodal melanins, which could be assigned to reduction processes. Tentatively, those peaks could be assigned to cation uptake processes because they appear in the same potential range in the absence of melanin. Nevertheless, the shape of the reduction wave points towards a reduction process involving hydroquinone, semiquinone, and quinone chemical structures identified previously in both melanins by EPR at 3524 G (**Figure 5**_right). These oxidative species, especially semiquinone, can act as a sink for unpaired electrons, explaining the quenching activity of melanin on free radicals (Dadachova et al., 2008; Eom et al., 2019). It is interesting to note that, in the absence of GS, the voltametric peaks for redox couples of quinone groups exhibited a very reversible behavior, in contrast with what is observed in the case of melanin/GS-coated electrodes. The explanation for this phenomenon can be found in the electroactive behavior of GS. At a potential of about −0.3V, GS can use electrodes as electron acceptors in its metabolism (Estevez-Canales et al., 2015). If quinones are involved in facilitating the extracellular electron transfer processes of GS, a reduction in quinones could be prevented when using GS as the electrode in an electron acceptor. Only when GS interacts with the electrode as an electron donor can the reduction in quinones be observed. Consequently, this process could explain the origin of the non-reversible behavior of the oxidation/reduction of quinone groups.

These results demonstrate that both types of melanins (fungal allomelanin and animal eumelanin) enhanced the bioelectroactivity of *Geobacter sulfurreducens*, despite their inherent chemical–structural differences. This phenomenon is likely due to the ability of melanin to act as a semiconductor, facilitating electron transfer between the organism and the electrode. The semiconducting nature of melanin holds promise for biotechnological applications, particularly in interspecies electron transfer and bioelectrode development (Kim et al., 2020).

### 3.4 Melanin–metal adsorption mechanisms

**Figure 7:**
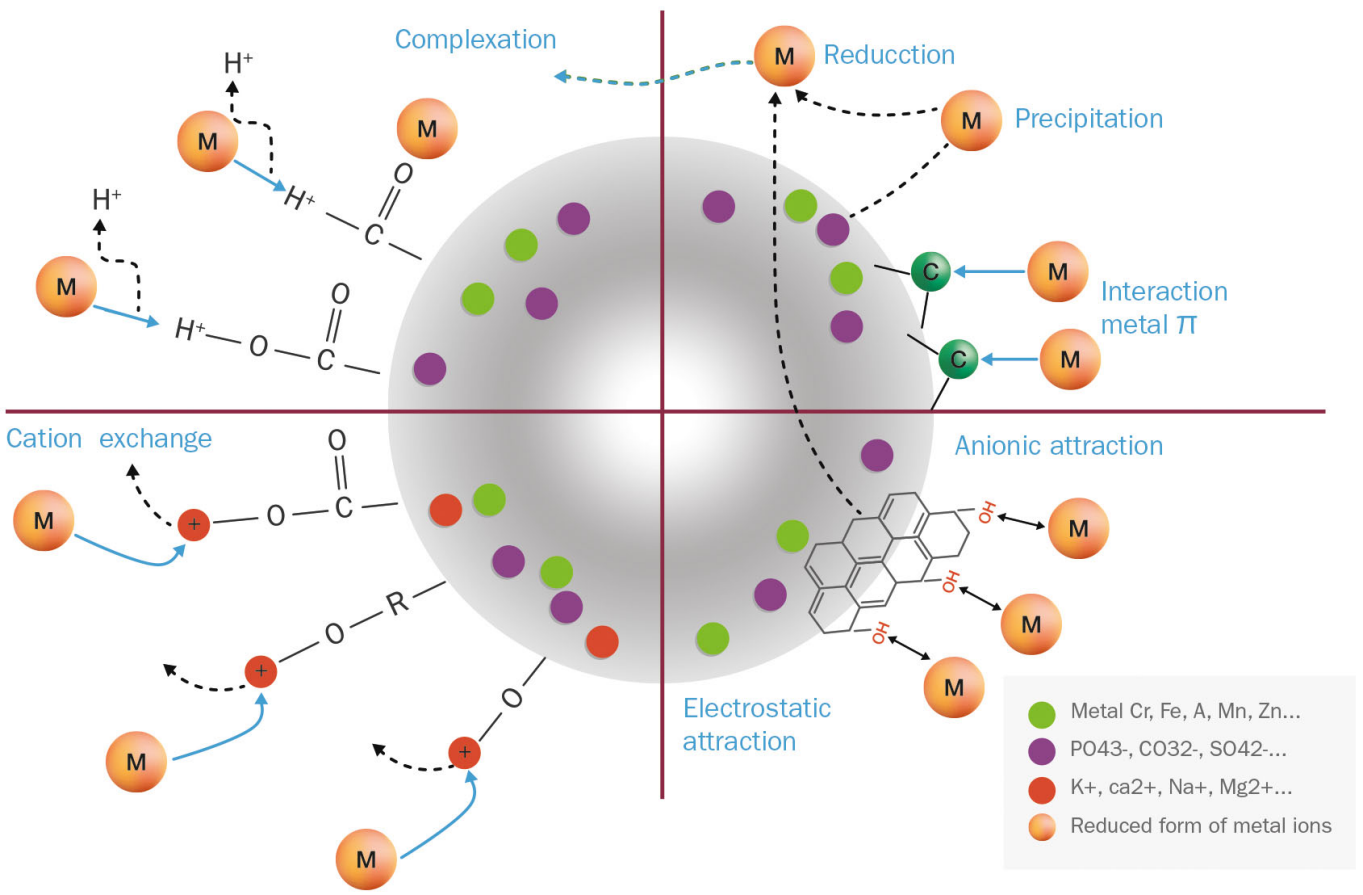
Proposed binding mechanisms of free Cr(VI) ions on an idealized melanin particle.

Samples of fungal and cephalopodal melanin extracts exposed to Cr(VI) were also analyzed by FTIR spectrometry (**Figure 5**). From the FTIR profiling of Cr(VI)-bound melanin extracts, it can be inferred that this phenomenon is governed by multiple processes, as illustrated in **Figure 7**. The obtained FTIR spectral profiles reveal enhanced vibrations in fungal melanin at 3499 and 1072. cm^−1^, suggesting the involvement of aromatic, COO^-^, and OH groups in Cr(VI) adsorption (**Figure 5**). Hydrogen bonding plays a primary role in the polarization of Cr(VI) ions, so theses peaks might be attributed to the attachment of Cr(VI) to OH groups (Kulkarni et al., 2013). The peak shift towards higher wavenumbers indicates the chemisorption of heavy metals onto the functional groups of melanin (Manirethan et al., 2018, 2020). The reduced intensity of the peaks at 2924 and 2854 cm^−1^ suggests a significant weakening of C–H vibrations due to the interaction between melanin and adsorbed Cr(VI).

The FTIR spectroscopic analysis of Cr(VI)-exposed melanin unveils intriguing alterations in its spectral profile. Specifically, the disappearance of absorption bands near 1650 cm^−1^, previously attributed to the quinone C=O stretching of carboxylic groups (COOH) resulting from oxidation, implies a significant change upon exposure to Cr(VI). Notably, fungal melanin exhibits a discernible decrease in C=O transmittance alongside the emergence of a distinct peak around 1550 cm^−1^ upon Cr(VI) exposure. This shift towards lower wavenumbers likely stems from the augmented electron density induced by Cr(VI) binding to adjacent functional groups within the melanin structure. This observed phenomenon resonates with established findings (Larsson, 1993; Manirethan et al., 2020). Furthermore, in contrast to treatments devoid of metal exposure, where various bands manifest in this region, the singular presence of this peak in Cr(VI) treatments suggests a complexation with the C=O functional group. The observed peak shift concurs with the notion of heightened electron density attributable to metal binding. This observation could also be attributed to precipitation and reduction processes occurring during the Cr(VI)–melanin interaction, as stated by Yang et al. (2023). The C=O stretching band serves as a dual indicator: firstly, for inferring the strength of the π bond within the carbonyl group, and secondly, for monitoring the Cr(VI) uptake process involving this functional group. These data suggest that Cr(VI) binding amplifies the electrostatic capacitance of melanin, a property that has been confirmed earlier through electrochemical characterization (Eom et al., 2019). This unique capacitance allows melanin to act as a semiconductor-like reservoir, facilitating the capture of unpaired electrons, as illustrated in **Figure 7**.

As for the sepia melanin (**Figure 5**), the shift of the OH absorption peak from 3105 cm^−1^ suggests the involvement of the carboxylic/phenolic hydroxyl group in the adsorption of Cr(VI). Alterations in the region 1240–1200 cm^−1^ further point to the role of the amine group in the adsorption process with this type of melanin. This observation aligns with previous reports (Liu et al., 2004; Liu and Simon, 2005; Di Mauro et al., 2017), in which sepia melanin is identified as a DOPA-melanin that contains nitrogenated functional groups (NH), in contrast to the nitrogen-free DHN-melanin, which seems to be the predominant type in *E. mesophila*.

### 3.5 Adsorption of hexavalent chromium onto fungal melanin

**Figure 8:**
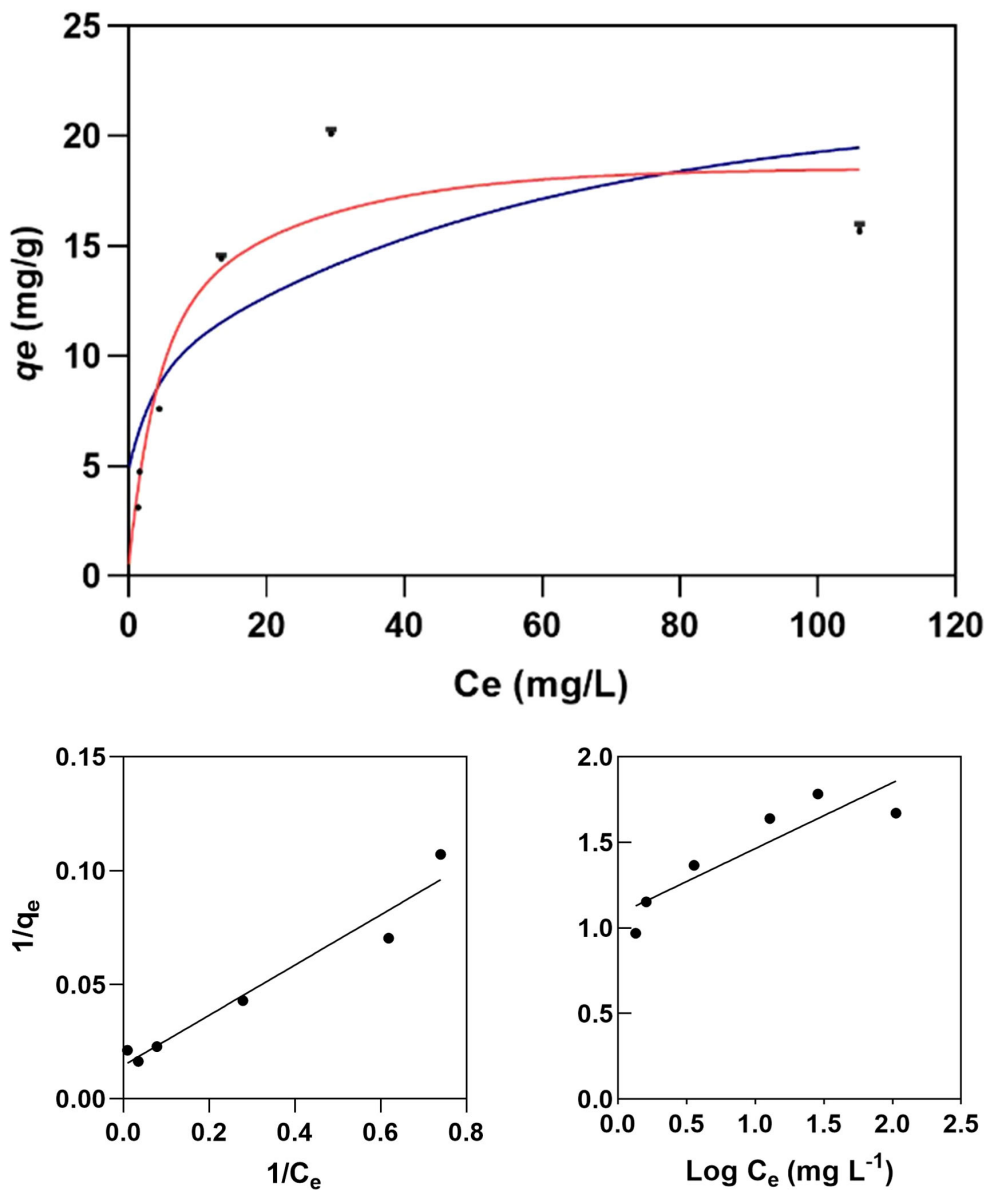
Langmuir (red line) and Freundlich (blue line) isotherm models for the adsorption of Cr(VI) on melanin (top graph) fitted to the experimental data (dots). Experimental conditions: initial Cr(VI) concentration 30 mg L^−1^, melanin content 6 g L^−1^, pH 6.5, temperature 25 °C.

The Langmuir isotherm model (**Figure 8**) provides a more accurate description of Cr(VI) adsorption by melanin extracts of *Exophiala mesophila* (R^2^ = 0.904) than the Freundlich isotherm (R^2^ = 0.670). **Table 1** summarizes the corresponding model parameters, in comparison with those from other similar studies. The Langmuir affinity constant seen here (*K_L_* = 0.193 L mg^−1^) was practically identical to the value we obtained previously by fitting experimental time-course adsorption data to a three-parameter model that integrated the Langmuir isotherm and second-order adsorption kinetics (Medina-Armijo et al., 2024). However, the modeled theoretical maximum Cr(VI) adsorption capacity obtained in this study (*q_max_* = 19.37 mg g^−1^) is lower than that which we obtained previously through dynamic experiments (*q_max_* = 95.26 mg g^−1^). Since equilibrium isotherms and kinetic models address different aspects of the adsorption process, the *q_max_* values they estimate might not necessarily be the same, as seen previously by other authors (Fahry et al., 2023). The higher correlation of experimental adsorption data to the Langmuir isotherm, compared to the Freundlich isotherm, suggests that fungal melanin extracts present a monolayer with a finite distribution of active sites on the surface for the adsorption of Cr(VI) ions. In similar previous studies (**Table 2**), values for the *q_max_* of melanin extracts ranged from 6.53 mg g^−1^ for sepia ink embedded in sodium alginate to 595.97 mg g^−1^ for crude melanin extracts of the black fungus *Aureobasidium pullulans*. However, these results must be analyzed and compared with caution, as the experimental conditions in which these assays have been conducted vary significantly, especially as regards the melanin content and extraction/functionalization procedure, as well as the pH, which both have a great impact on the adsorption process.

**Table 2:** The absorption of Cr(VI) onto the fungal melanin powder used here, based on Langmuir and Freundlich isotherm parameters, and comparison with the results of previous studies.

Source	Matrix	Langmuir isotherm			Freundlich Isotherm			pH	Dosage (g L^−1^)	Reference
		q_max_ (mg g^−1^)	K_L_ (L mg^−1^)	R^2^	K_F_ (mg g^−1^)	n	R^2^			
Fungal biomass (*Exophiala mesophila*)	Melanin extracts	19.37	0.19	0.904	5.96	3.94	0.670	6.5	6.0	This study
Fungal biomass (*Exophiala mesophila*)	Melanin extracts	95.26	0.20	0.924	-	-	-	6.5	6.0	Medina-Armijo et al. (2024)
Fungal biomass (*Aureobasidium pullulans*)	Melanin extracts	595.97	0.04	0.986	1.91	1.55	0.959	5.0	0.1	Fahry et al. (2023)
Cephalopodous ink (*Sepia officinalis*)	Melanin embedded in sodium alginate	6.53	0.11	0.955	0.79	2.04	0.989	2.0	20	Cuong et al. (2018)
Bacterial biomass (*Peudomonas stutzeri*)	Melanin extracts	126.90	0.30	0.99	28.93	1.69	0.97	3.0	0.2	Manirethan et al. (2018)

The parameter constants of the Freundlich isotherm *K_F_* and n also provide useful information for a better understanding of the adsorption behavior. *K_F_* corresponds to the Freundlich adsorption capacity, which reflects a combination of adsorption capacity and affinity, while the dimensionless *n* characterizes the heterogeneity of the system. For a viable adsorption process, the parameter *n* is usually greater than the unity, but the larger the *n* value is, the more heterogeneous the system is. When comparing this study with the limited literature on the melanin adsorption of Cr(VI) (**Table 2**), we find that the obtained *K_F_* = 5.96 mg g^−1^ and *n* = 3.94 indicate that melanin extracted from the black fungus *E. mesophila* has a relatively high adsorption potential, despite being a comparatively heterogeneous adsorbent as well.

In summary, melanin’s unique physicochemical properties—metal sorption capacity, resistance to acidic pH, and high-temperature stability—make it an attractive biomaterial for various environmental applications. Novel melanin-based nanoparticles have shown significant improvements in metal removal rates from polluted waters, compared to conventional methods (Tran-Ly et al., 2020b). Besides heavy metals, previous studies have shown melanin’s capacity to bind diverse pollutants, including organic compounds and synthetic dyes (Lino et al., 2022). Such specific sorption capacity presents a promising avenue for the development of highly sensitive and selective sensors for heavy metals and other environmental contaminants (Steven et al., 2007). Melanin’s electrochemical interactions with metal ions and other charged molecules offer new options for modulating electron transfer processes within microbial syntrophic communities that govern important bioprocesses such as methanogenesis (Sriphirom et al., 2021). The large-scale production of melanin and its regeneration after metal sorption are crucial for the economic viability and long-term sustainability of melanin-based treatments (Lino et al., 2022; Michael et al., 2023). Black fungi like *Exophiala mesophila* can easily be cultivated by common fermentation techniques, offering a new scalable source of melanin. However, the production of melanin for industrial applications also requires the optimization of melanin extraction techniques (Choi, 2021). While this study provides a preliminary insight into the characteristics and potential application of melanin from black fungi, further research efforts are warranted to develop efficient and cost-effective strategies for large-scale melanin production.

## 4 Conclusion

This study describes a detailed physicochemical characterization of melanin extracted from the metallotolerant black fungus *Exophiala mesophila* (Chaetothyriales). Based on inhibitory studies using the fungicide tricyclazole in relation to whole cells, as well as FTIR spectrometric analyses performed on melanin extracts in comparison to natural and synthetic DOPA-melanin, we conclude that this fungus produces melanin primarily via the DHN pathway. The electrochemical characterization of melanin extracts also demonstrated that this biomaterial exhibits semiconductor-like properties that may lead to interesting interactions between fungal melanin and other electroactive microorganisms. Based on these results and using Cr(VI) as a model heavy metal, we also conclude that the melanin–metal binding process primarily occurs through electrostatic interactions and cation exchange mechanisms. These findings pave the way for the development of novel biotechnological applications utilizing fungal melanin. Potential areas of exploration include the bioremediation of environmental pollutants, sensor development, nanoparticle synthesis, and bioelectrochemical processes.

## Data availability statement

The raw data supporting the conclusions of this article will be made available by the authors, without undue reservation.

## Author contributions

CM-A: Conceptualization, Data curation, Formal analysis, Investigation, Methodology, Software, Writing – original draft, Writing – review & editing. IY: Methodology, Validation, Project administration, Writing – review & editing. AB: Methodology, Validation, Writing – review & editing. AP: Methodology, Validation, Writing – review & editing. AE-N: Methodology, Validation, Writing – review & editing. MV: Funding acquisition, Methodology, Project administration, Validation, Writing – review & editing. FP-B: Conceptualization, Project administration, Supervision, Writing – review & editing.
